# Ca2+ fortified oral rehydration solution is effective in reducing diarrhea morbidity in cholera toxin-pretreated mice

**DOI:** 10.21203/rs.3.rs-3482753/v1

**Published:** 2023-10-29

**Authors:** Lieqi Tang, Shi Jin, Steven Winesett, Jane Harrell, Johnathan Fraebel, Sam X. Cheng

**Affiliations:** University of Florida; University of Florida; Malcom Randall VA Medical Center; University of Florida School of Medicine; University of Florida School of Medicine; University of Florida

## Abstract

Diarrhea like cholera remains a leading cause of mortality and morbidity globally. Oral rehydration solution (ORS) that developed in 1970s significantly decreases diarrhea mortality; yet, it does not reduce diarrhea morbidity and its usage has reduced persistently. Patients with diarrhea lose not only monovalent ions Na^+^, K^+^, Cl^−^ and HCO_3_, which are replaced via ORS, but also divalent ions Zn^2+^ and Ca^2+^, which are not routinely replaced, particularly for Ca^2+^. Using several *in vitro* technologies performed in isolated tissues, we have previously shown that Ca^2+^, a primary ligand that activates the Ca^2+^-sensing receptor, can act on intestinal epithelium and enteric nervous system and reverse cholera toxin-induced fluid secretion. In the present study, using the cholera toxin-pretreated C57BL/6 mice as a model, we show that the anti-diarrheal effect of Ca^2+^ can also occur *in vivo*. Our results raise a question of whether this divalent ion also needs to be replaced in diarrhea management. Perhaps, an ideal rehydration therapy would be solutions that contain both monovalent ions, which reduce diarrhea mortality, and divalent minerals, which reduce diarrhea morbidity.

## INTRODUCTION

Diarrhea disease such as cholera remains a leading cause of mortality and morbidity among the most vulnerable populations^[1]^, accounting for approximately 9 per cent of all deaths among children under age 5 worldwide in 2019^[2]^ and 2.39 billion estimated cases of diarrhea in 2015^[3]^. Despite recent advances in research from molecular biology to public health initiatives, few advancements have been made in the realm of diarrhea therapeutics, and oral rehydration solution (ORS) developed in the early 1970s has remained the mainstay therapy. Although use of ORS has decreased the diarrheal mortality, it does not reduce its morbidity^[4]^. ORS neither reduces disease severity nor duration and its usage has declined persistently^[5]^. Currently, only one third of cases are used^[5]^ for many reasons but most notably due to perceived failure to reduce patient’s stool output. Therefore, for decades there has been continuous effort searching for new therapeutics that are able to halt diarrhea and alleviate disease symptoms^[5–7]^, particularly those simple, widely available and inexpensive therapeutics^[4, 8–10]^.

Patients with diarrhea lose not only monovalent ions (e.g., Na^+^, K^+^, Cl^−^ and HCO_3_^−^) but also divalent minerals (e.g., Zn^2+^ and Ca^2+^, which are not routinely replaced)^[11, 12]^. While losses of monovalent ions are replaced via ORS, losses of divalent minerals are not. Studies now suggest that despite divalent minerals contributing relatively few osmoles to diarrhea stool, their effects on intestinal functions are large due to the presence of divalent mineral-sensing receptors (e.g., Zn^2+^-sensing receptor, ZnSR^[13]^; Ca^2+^-sensing receptor, CaSR^[14]^) in the gut and their amplifying effects. As a result, losses of Zn^2+^ from diarrheal stools without replacement will significantly increase the magnitude and duration of diarrhea symptoms; replacements of Zn^2+^ losses will reduce the severity of diarrheal illnesses and accelerate their recovery. This is well documented and today Zn^2+^ replacement has been adopted as policy in all countries^[15–18]^. In 2019, World Health Organization has added user-friendly Zn^2+^/ORS co-pack to the Essential Medicine List^[19]^.

Relative to loss of Zn^2+^, which is 3.4x normal, the loss of Ca^2+^ in diarrhea is far more severe, at 20.5x normal loss^[11, 12, 20]^. Given the presence of CaSR in the intestine^[21, 22]^ and its ‘all-inclusive’ antidiarrheal effects shown in recent studies^[21–31]^, a question arises of whether this divalent ion lost in diarrhea also needs to be promptly replaced^[4, 20, 32, 33]^. We hypothesized that, like Zn^2+^, without correcting the negative Ca^2+^ balance, diarrheal disease would be more severe and more protracted; with Ca^2+^ replacement, diarrhea severity and duration would be reduced. Perhaps, an ideal ORS would contain both monovalent ions and divalent minerals, particularly Ca^2+^. With Ca^2+^-fortified ORS, we anticipate that diarrhea severity will be reduced, duration shortened, and recovery accelerated.

Therefore, this present study compared ORS with *vs*. without Ca^2+^ in reducing cholera toxin-induced watery diarrhea in experimental C57BL/6 mice as a critical step before randomized controlled trials are conducted in humans. *Vibrio cholerae* produces diarrhea via cholera toxin (CTX). We employed CTX mouse model of secretory diarrhea not only because most of our prior preclinical studies *in vitro* were performed using this model, including CaSR knockout mouse studies^[24–28]^, but also because this well characterized enterotoxin secretagogue has been widely used as a human alternative to provide proof of concept of whether an antidiarrheal agent is therapeutic or not ^[34–37]^. We did not use the bacteria *Vibrio cholerae* as a model primarily for concerns of known confounding effects of the Ca^2+^ on bacterial colonization^[38]^ and biofilm formation^[39]^ that would complicate result interpretation. To make certain that the anti-diarrheal effect was specific to the Ca^2+^, not due to the associated anion of Ca^2+^ compounds, we tested three different sources of Ca^2+^, i.e., Ca^2+^ citrate, Ca^2+^ chloride, and Ca^2+^ carbonate. A drug effect is a function of time and dose; to further demonstrate the specificity of the effect of the Ca^2+^, we also studied Ca^2+^ time and dose responses. Our results show that Ca^2+^ supplemented ORS is indeed reducing diarrheal morbidity, at least in cholera toxin-pretreated mice.

## RESULTS

### ORS containing Ca^2+^ citrate is more effective than standard ORS in reducing the severity of cholera toxin-induced diarrhea

**Figure 1:**
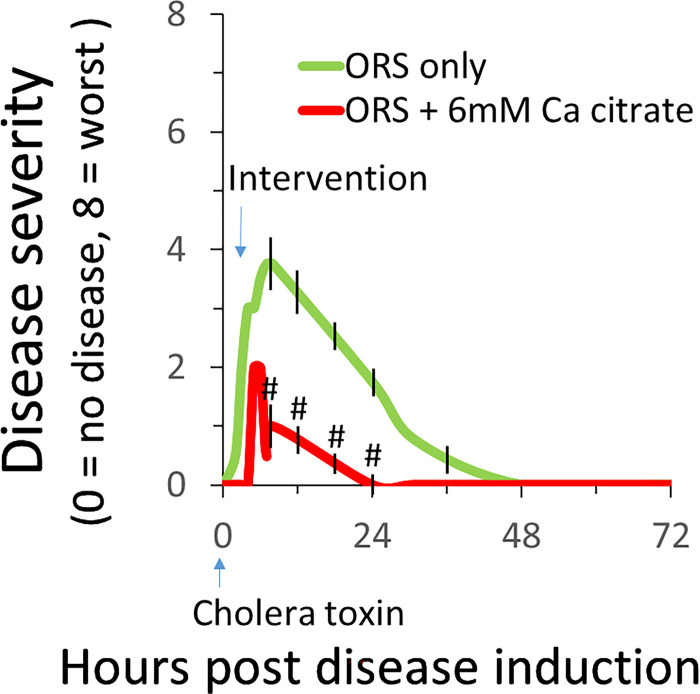
ORS containing Ca^2+^ citrate is better than standard ORS in reducing cholera toxin-induced diarrhea disease. At hour 0, all animals were intragastrically gavaged with 10 μg/mouse of cholera toxin to induce diarrhea disease. At hour 1.5, animals were assigned to receive water bottles that contain ORS alone or ORS with 6 mM Ca^2+^ citrate for 72 hours. Diarrheal disease was monitored and disease severity scored every 2 hours in day 1 and every 12 hours thereafter. Shown are means ± SE. n=7. #P<0.05 *vs*. ORS only. For clarity, only those means whose differences were statistically significant were shown with SE. Without CTX treatment, all mice did not exhibit disease activity regardless they received ORS alone or ORS with Ca^2+^ (not shown).

In Experiment 1, cholera toxin (10 μg/mouse) was gavaged into the stomach of 6–8-week-old young adult C57BL/6 mice to first induce diarrheal disease. Ninety minutes later when diarrhea formed, mice were randomly assigned to receive ORS alone (control) or ORS with 6 mM Ca^2+^ citrate (intervention) for 3 days, and changes in disease activity were monitored and diarrhea severity scored according to Table 1. Figure 1 shows time-dependent changes in disease activity score and their responses to ORS *vs*. ORS containing Ca^2+^.

In control group, the cholera toxin treatment induced moderately severe diarrheal disease. The diarrhea disease started to occur ~ 2 hours after the gavage of the toxin, peaked at 8 hours, then slowly recovered. The disease did not completely recover until 36–48 hours post disease induction. In intervention group, although mice receiving Ca^2+^ also developed diarrheal disease within 2 hours and peaked at 6–8 hours, they developed a significantly less severe and less pronounced disease. The disease activity scores mean ± SE (n), expressed as AUC (area under curve) control *vs*. intervention: 81.2 ± 1.6(7) *vs*. 14.3 ± 1.9(7) (P < 0.05). The disease duration (hours): 34.6 ± 1.2(7) *vs*. 14.5 ± 1.4(7) (P < 0.05). Ca^2+^ reduced diarrhea severity and duration by 82% and 58%, respectively. Thus, while in ORS treated mice the disease activity score had remained significantly elevated above baseline until 36–48 hours post CTX exposure, in ORS + Ca^2+^ treated mice a close to normal activity score had been achieved at least 20 hours earlier at 12–24 hours post CTX treatment (Fig. 1).

### ORS containing Ca^2+^ chloride is more effective than standard ORS in reducing the severity of cholera toxin-induced diarrhea

**Figure 2:**
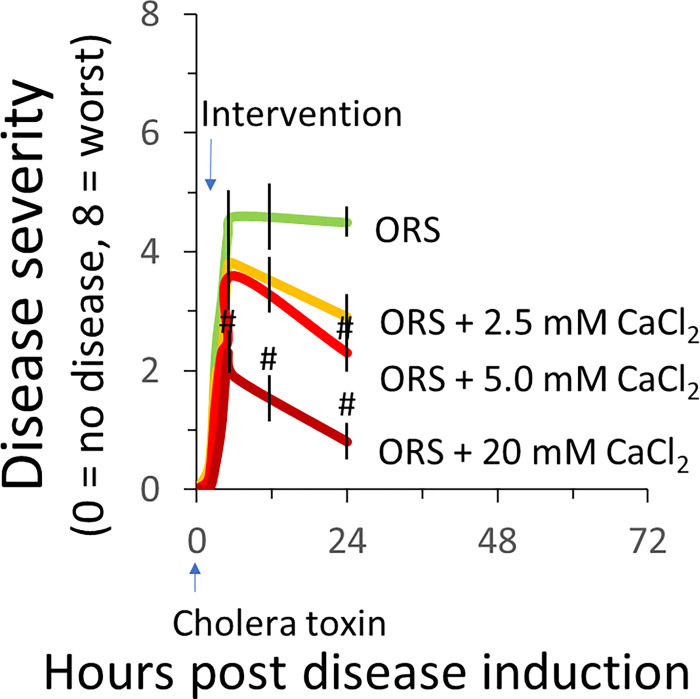
ORS containing Ca2+ chloride is better than standard ORS in reducing cholera toxin-induced diarrhea disease. At hour 0, all animals were intragastrically gavaged with 10 μg/mouse of cholera toxin to induce diarrhea disease. At hour 1.5, animals were assigned to receive water bottles that contain ORS alone without Ca2+ or ORS with 2.5, 5 or 20 mM Ca2+ chloride for 24 hours. Diarrheal disease was monitored and disease severity scored. Shown are means ± SE. n=5. #P<0.05 *vs*. ORS only.

To be certain that the observed anti-diarrheal effect was specific to the Ca^2+^ not due to the anion, in Experiment 2, we compared the effects of ORS with Ca^2+^ chloride *vs*. standard ORS. The same protocol and treatments used in Experiment 1 were employed. In this experiment, we also examined the dose effect of the Ca^2+^. Figure 2 shows CTX-induced changes in disease activity in response to ORS with 0–20 mM Ca^2+^ chloride. Ca^2+^ reduced diarrhea; the higher concentration of the Ca^2+^ in ORS, the lower activity score of the diarrhea. The disease activity scores at 24 hours’ treatment with ORS + Ca^2+^ 0 (control), 2.5, 5 & 20 mM mean ± SE (n) (P value *vs*. control): 4.3 ± 0.2(5) (control), 3.0 ± 0.3(5) (P > 0.05), 2.5 ± 0.3(5) (P < & 0.8 ± 0.3(5) (P < 0.05). Thus, diarrhea reduced by 35% at low [Ca^2+^] of 2.5 mM, 50% at medium [Ca^2+^] of 5 mM, and 80% at high [Ca^2+^] of 20 mM.

### ORS containing Ca^2+^ carbonate is more effective than standard ORS in reducing the severity of cholera toxin-induced diarrhea

**Figure 3:**
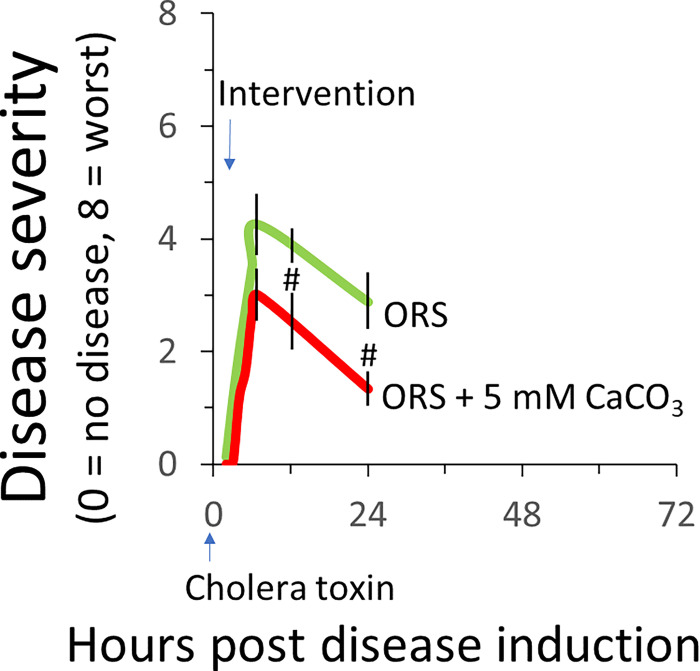
ORS containing Ca^2+^ carbonate is better than standard ORS in reducing cholera toxin-induced diarrhea disease. At hour 0, all animals were intragastrically gavaged with 10 μg/mouse of cholera toxin to induce diarrhea disease. At hour 1.5, animals were assigned to receive water bottles that contain ORS alone without Ca^2+^ or ORS with 5 mM Ca^2+^ carbonate for 24 hours. Diarrheal disease was monitored and disease severity scored. Shown are means ± SE. n=5. #P<0.05 **vs*.* ORS only.

To further confirm the Ca^2+^ effect, in Experiment 3, we tested Ca^2+^ carbonate using the same disease model and treatment protocol. Figure 3 shows disease responses to ORS without *vs*. with 5 mM Ca^2+^ carbonate. Compared to mice who drank ORS without Ca^2+^, mice who drank ORS with Ca^2+^ had a significantly less severe disease.

Taken together, these results demonstrate that adding Ca^2+^ to ORS, regardless of whether the Ca^2+^ added is Ca^2+^ citrate, Ca^2+^ chloride or Ca^2+^ carbonate, all reduces diarrhea.

## DISCUSSION

This is the 1st *in vivo* demonstration, showing that Ca^2+^ fortified ORS is more effective than standard ORS in reducing diarrheal morbidity. This includes reducing not only the severity but also the duration. The finding is in keeping with the findings in Zn^2+^ research, suggesting that both mono- and di-valent ions lost in diarrheal stools should be promptly replaced during rehydration therapy. Inadequate replacement of these divalent ion losses may lead to a more severe and more prolonged course of the diarrhea.

Table 2 summarizes the losses of ions in diarrhea and their health impacts as well as our proposed clinical treatments. According to this, both monovalent ions (Na^+^, K^+^, Cl^−^, and HCO_3_^−^) and divalent minerals (Ca^2+^, Zn^2+^) are lost that can impact diarrhea outcomes. Without replacing monovalent ions, patients with diarrheal stools may develop hypovolemia, hyponatremia, hypokalemia, hypochloremia and metabolic acidosis, increasing risk of diarrheal mortality; without replacement of lost divalent minerals, the body will develop Ca^2+^ deficit/CaSR hypofunction and Zn^2+^/ZnSR hypofunction, leading to increase in diarrheal morbidity, making the disease more severe and more protracted. The proposed solution for the former is ORS rehydration/replacement therapy, which is well recognized and has been standard of care. Whereas the clinical solution for the latter is to add back Ca^2+^/Zn^2+^, which is still not fully recognized, particularly for replacing of Ca^2+^.

We showed that, without Ca^2+^ replacement in their drinking ORS bottles, mice developed a significantly more severe and more protracted diarrheal disease when exposing to cholera toxin provocation; with Ca^2+^ replacement, the disease severity was significantly reduced so was the time to diarrhea recovery (see Fig. 1). We further showed that the antidiarrheal effect observed was due to the Ca^2+^ but not the associated anions because no matter what the Ca^2+^ compound was employed, it inhibited diarrhea. This applied to Ca^2+^ citrate (Fig. 1), Ca^2+^ chloride (Fig. 2), and Ca^2+^ carbonate (Fig. 3).

How does Ca^2+^ ameliorate CTX-induced diarrhea? CTX as well as other enterotoxins produces diarrhea through direct epithelial action (including trans- and para- epithelial action) and indirectly by activating enteric nervous system (ENS)^[40, 41]^. According to our previous work, Ca^2+^ can act via CaSR and reverse the diarrhea-producing pathways provoked by CTX. For example, while CTX activates adenylyl cyclase and cyclic AMP generation, Ca^2+^/CaSR activates phosphodiesterase and cyclic nucleotide destruction^[24]^. Similarly, while CTX reduces claudin-2, opening up tight junctions, Ca^2+^/CaSR enhances claudin-2, closing down tight junctions^[25]^. Finally, while CTX activates the ENS-mediated secretion, Ca^2+^/CaSR inhibits it^[28]^. However, considering that Ca^2+^/CaSR enhances degradation but does not prevent generation of the 2nd messengers induced^[24]^, Ca^2+^ is not anticipated to prevent occurrence anticipated to prevent occurrence of diarrhea although this latter was not explored in the present study.

The present study used simultaneous provision of the two effective interventions Ca^2+^ and ORS. We did it both for physiological and clinical considerations. Physiologically, this combined approach would maximally increase the Ca^2+^ bioavailability thus greatest stimulates the CaSR in the intestine. Diarrhea with high stool output will speed up the passage of substances through the digestive tract, particularly if they were delivered in tablets or boluses^[42]^. This will inevitably reduce the residence and contact time of the substances with the intestine unless they are delivered frequently and or in high doses. Otherwise, their anticipated drug effect on diarrhea may be compromised or not produced as evidenced in a recent study with an oral CFTR inhibitor^[43]^. Continuously or frequently administering Ca^2+^ via ORS in a drinking bottle that was adopted in the present study could avoid or minimize this risk.

The provision and administration of the two effective interventions Ca^2+^ and ORS together will also have clinical significance, particularly in remote areas of developing countries, where there are health system obstacles to widespread use of a separate Ca^2+^ as well as Zn^2+^ during acute diarrhea. These obstacles include the need for Ca^2+^/Zn^2+^ suspension, syrup or tablets to be made available at health facilities and prescribed by health care providers in addition to ORS. Thus, if Ca^2+^ and ORS can be co-delivered or co-packaged together, it could considerably increase treatment prescription, administration, compliance, and adherence of the both interventions. In addition, it could simplify the logistics of incorporating Ca^2+^ into treatment guidelines for diarrhea, as does for Zn^2+^ and ORS^[19]^.

In summary, diarrhea remains a leading cause of mortality and morbidity globally. While ORS helps reduce diarrheal mortality, it does not reduce diarrheal morbidity. We show that adding Ca^2+^ to ORS might be a solution to help reducing diarrheal morbidity. If this is verified in humans, this simple approach could benefit hundreds of millions of diarrhea patients worldwide. Randomized controlled trials in humans are under way.

## METHODS

### Animals.

Experiments were performed using 6–8 weeks of age male/female C57BL/6 mice that were bred and maintained in-house at the University of Florida Communicore Animal Facility in accordance with the Animal Welfare Act and the Public Health Policy on Humane Care. Animals were fed and maintained on regular chow (Harlan) with free access to water before experiment. After completion of the experiment, animals were sacrificed with standard CO_2_ inhalation and by cervical dislocation. The use of animals as well as the protocols for cholera toxin and ORS treatments was approved by the Institutional Animal Care and Use Committee (IACUC# 201807567) at University of Florida.

### Cholera toxin mouse model of secretory diarrhea.

Animals were first fasted 16 hours before they were gavaged, intragastrically, with 200μl 7% NaHCO_3_ buffer containing 10μg CTX. In these experiments, 10μg CTX per mouse was dosed to induce a moderate severity of diarrheal disease. After CTX gavage, animals continued fasted for additional 90 minutes before they were allowed access to drinking water (i.e., ORS with or without Ca^2+^) and regular chow in order to avoid their possible interferences on toxin binding and action. Our pilot studies showed that diarrhea started ~½ hour and peaked ~ 1½ hours post cholera toxin gavage consistent with a previous report^[44]^. To reduce bias, animals were assigned, randomly, to receive ORS with *vs*. without Ca^2+^ by one investigator and then monitored for progression of disease, blindly, by different investigators. Previously, we scored disease activity using stool consistency score; while it is simpler, stool consistency score was often found difficult to correctly evaluate the diarrhea severity, particularly when the stool was stuck to the bedding chips. Also, it was hard to appropriately determine the exact grade for the parameter owing to the acute nature of watery diarrhea induced and the overlapping ambiguous boundaries between grades. Consequently, in this present study, we employed the disease activity score that uses sum of stool consistency, appearance and activity as an indicator of health (Table 1). The disease activity score was calculated by the sum of scores assigned for each category (stool consistency, appearance, and activity) based on scoring system (Table 1). This scoring technique has been shown to be more accurately reflecting the wellbeing and state of health of mice^[45]^. Moreover, it is less sensitive to inter-observer variability^[45]^.

### Chemicals and solutions.

Cholera toxin was obtained from Sigma-Aldrich, and 5 mg/ml stock solutions were prepared in water. Sodium chloride, potassium chloride, sodium citrate, citric acid, Ca^2+^ chloride, Ca^2+^ citrate, Ca^2+^ carbonate and glucose were all purchased from Sigma-Aldrich. ORS was prepared freshly containing (in mM) 75 Na^+^, 20 K^+^, 65 Cl^−^, 10 citrate and 75 glucose with total osmolarity of 257 mOsm/kg H_2_O.

### Statistical Analysis.

Values are given as means ± SEM. Statistical comparisons between two means were performed by Studenťs t-test, whereas comparisons among multiple means were by one-way ANOVA with Tukey’s post hoc tests. *P* < 0.05 was considered significant.

## Figures and Tables

**Table 1:** Scoring system for indicators of disease severity in mice

Stool consistency	Score
Normal	0
Pasty	1
Watery	2
Perianal stain	3
Appearance	
Normal	0
Piloerection	1
Hunched back	2
Activity	
Normal	0
Suppressed	1
Move when provoked	2
Not move when provoked	3

**Table 2:** Losses of ions in normal infants and those with diarrhea, their consequences and proposed interventions.

Loss	Normal	Diarrhea	Fold increase	Consequences	Outcomes	Treatment recommendations
Water	81	162	2.0	Hypovolemia		
Na^+^	2.15	12.37	5.7	Hyponatremia		
K^+^	4.15	4.92	1.2	Hypokalemia	↑ Mortality	ORS
Cl^−^	2.69	9.36	3.5	Hypochloremia		
HCO_3_^−^	3.61	7.91	2.2	Metabolic acidosis		
Ca^2+^	0.16	3.27	20.5	Ca^2+^ deficit◊↓CaSR	↑ Morbidity	ORS + Ca^2+^
Zn^2+^	0.00072	0.00243	3.4	Zn^2+^ deficit◊↓ZnSR		ORS + Zn^2+^

The water loss is expressed in gram/kg body weight/day, whereas ion losses are in milli-mole/kg body weight/day. The calculation of Zn^2+^ loss is based on the data obtained from a metabolic balance study in human infants^[12]^, whereas the calculations of other losses are based on the data from a metabolic balance study in infant calves^[11]^. The loss of HCO_3_^−^ is estimated by subtracting the Cl^−^ loss from the combined Na+ and K+ losses. Note that, unlike monovalent ions, divalent ions have additional impacts on physiologic processes besides their established nutritional functions. These include the activation of extracellular calcium-sensing receptor (CaSR) by Ca^2+^ and the activation of extracellular zinc-sensing receptor (ZnSR) in the intestine by Zn^2+^.

## Data Availability

The datasets generated during and/or analyzed during the current study are available from the corresponding author on reasonable request.
